# Supplementary material for: Revealing CO2 dissociation pathways at vicinal copper (997) interfaces
Source: Nat Commun. 2023 Jun 6;14:3273. doi: 10.1038/s41467-023-38928-1 (PMC10244362; doi:10.1038/s41467-023-38928-1)
Supplement: Supplementary file 1 — Supplementary Information [file 41467_2023_38928_MOESM1_ESM.pdf]

Supplementary Information for

# Revealing $CO_2$ dissociation pathways at vicinal copper (997) interfaces

Jeongjin Kim[1,†], Youngseok Yu[2,3], Tae Won Go[1], Jean-Jacques Gallet[4,5], Fabrice Bournel[4,5], Bongjin Simon Mun[2,3,*], and Jeong Young Park[1,*]

[1]Department of Chemistry, Korea Advanced Institute of Science and Technology (KAIST), Daejeon 34141, Republic of Korea.

[2]Department of Physics and Photon Science, School of Physics and Chemistry, Gwangju Institute of Science and Technology (GIST), Gwangju 61005, Republic of Korea.

[3]Center for Advanced X-ray Science, GIST, Gwangju 61005, Republic of Korea.

[4]Laboratoire de Chimie Physique-Matière et Rayonnement, CNRS, Sorbonne Université, Paris 75005, France.

[5]Synchrotron SOLEIL, Saint-Aubin, Gif sur Yvette 91192, France.

[†]Present address: Chemistry Division, Brookhaven National Laboratory, Upton, New York 11973, United States.

[*]E-mail addresses: bsmun@gist.ac.kr (B.S.M); jeongypark@kaist.ac.kr (J.Y.P.).

**The PDF file includes:**

1. **Supplementary Text**
2. **Supplementary Figures 1–16**
3. **Supplementary References**

## 1. Supplementary Text

**Probing vicinal morphology on the metallic surface using the STM tip.** The vicinal (high-Miller-index) surface is susceptible in comparison with the low-Miller-index surface. In the straightforward surface cleaning procedure, all noble gas sputtering parameters (applied voltage, ion-dose amount, and duration time) affect the surface roughness of the vicinal planes[1]. Crystal annealing parameters, such as temperature and retention time, could also be critical to preparing well-defined vicinal surfaces. For example, the intense $Ar^+$ ion-bombardment sputtering process can form deep pits and holes on the Cu(997) surface. But the annealing process cannot heal the damaged regions quickly at mild temperatures. That may lead to the formation of reconstructed micro-facets, consisting of the triangular zig-zag structure, by activating the Schwoebel–Ehrlich barrier[2]. In this case, the experimental temperature determines surface diffusion and phase transition following the phase diagram of condensed matter[3]. A step-flow model can describe the order of metallic atoms' kinetic motion at the step and terrace sites.[4]

$$V = \frac{D_s}{\Delta c}\{\nabla c|_+ - \nabla c|_-\} \cdot \hat{n} = V_+ + V_- \quad \cdots\cdots\cdots \text{Eq. (1)}$$

Eq. (1) determines the diffusion velocity of the corresponding step sites at steady-state ($D_s$: Adatoms diffusion on the terrace, $\Delta c$: the atomic density difference between the quantity of terrace and adjacent step, $n$: a unit of normal diffusion along a perpendicular direction of step).

Observed frizzy STM images are related to the weak interaction between a scanning STM tip and defects on the surface. This situation is frequently observed at step-edges in the scanning area. The breaking away motion of metal atoms at the step-edges can be described as the relationship between the tunneling distance and scanning tip speed[5]. In the meantime, unwanted tip−surface contacts or strong attractions can generate a few kinks at the step-edges, depending on STM observation parameters. Notably, the dynamic diffusion of surface atoms is noticeable at roughened step sites of coinage materials such as Au[6], Cu[7], and Ag[8]. The escape of Cu atoms from the step-edges would be significantly decreased under the CO(g) environment because of the CO-Cu binding. Therefore, we can exclude the tip-induced surface restructuring issue at elevated CO pressures. The step-broken clustering on the Cu(997) surface was reproducibly observed in different scanning areas.

**Catalytic behaviors of dissociated O and CO adsorbates from $CO_2$.** Comparison results probed with AP-XPS and AP-STM exhibit the observed catalytic $CO_2$ activation over vicinal Cu surfaces in

order to elucidate $CO_2$ dissociation and further interactions with dissociated oxygen and CO adsorbates at Cu step-edge sites under gaseous $CO_2$ environments. In Supplementary Fig. 16, the plotted photoelectron signals in Cu 2*p*, O 1*s*, and C 1*s* core-level spectra (bottom; black line) were collected from the as-cleaned Cu(997) surface in UHV. They are consistent with the synchrotron-based AP-XPS results of the clean vicinal Cu surfaces. Although we characterized a small adventitious carbon feature at 284.5 eV after repeating surface cleaning cycles in UHV, we could not find any critical relationship between the adsorbed carbon species and dissociated adsorbates during the AP-XPS measurements under 0.8 mbar $CO_2$(g) conditions.

Unlike AP-XPS characterizations on Cu surfaces under CO(g) conditions, we find versatile adsorbate species in O 1s and C 1s core-level AP-XP spectra under the 0.8 mbar $CO_2$(g) conditions (middle; red line). In addition, the representative peak of gas-phase $CO_2$ clearly shows up in both spectra. The deconvoluted peaks at 529.9 and 531.1 eV could be attributed to adsorbed atomic O and O-C-O*/C-O* related species. Their correlated characterization results in the C 1*s* core-level AP-XP spectrum support that the resolved peaks at 286.2 and 288.8 eV are close to the fingerprint regions of Cu-CO and carbonate, formate, or carboxylate species ($CO_2^{\delta-}$)[9] in AP-XPS analyses, respectively. Still, the portion of labeled C* species in the C 1*s* peak deconvolution has negligible changes compared to the same feature measured in UHV.

After gas evacuation, the evolved features at 529.6–531.4 eV remain in the O 1s core-level spectrum, which would be associated with adsorbed oxygen[10,11] on the Cu(997) surface. Even though the characterized peak region may overlap with the well-established hydroxyl group-containing species at 530.8–531.3 eV[12,13], the water contamination was not identified in the same spectrum. In addition, the characterized Cu 2*p* core-level AP-XP spectrum verifies no significant change in Cu $2p_{3/2}$ and Cu $2p_{1/2}$ peaks before and after introducing $CO_2$ gas molecules. We note that the transient evolution of satellite features at 943–948 eV in the Cu 2*p* core-level AP-XP spectrum is only found under 0.8 mbar $CO_2$(g) conditions. These unusual features in our present study are not relevant to the representative shake-up peaks of Cu(I) or Cu(II) oxides. They clearly disappear from the Cu 2*p* spectrum after $CO_2$(g) evacuation during the lab-based AP-XPS measurements.

From the collected AP-XP spectra and corresponding morphology images under similar experimental conditions, we could propose the role of dissociated oxygen and CO adsorbates from $CO_2$ gas molecules on the vicinal Cu surface. Although the $CO_2$ molecule has a very low chance of effective collisions at the gas-solid interface, the stepped Cu edges can offer active sites for $CO_2$ activation at ambient pressures. As a result, the dissociated O* and CO* would have an interplay

between their adsorption sites and the formation of Cu nanoclusters along the stepped Cu geometry. The dissociated CO* adsorbates may induce step-broken Cu nanoclusters at Cu step-edge sites; in turn, they would evolve reversible surface restructuring at ambient pressures. In contrast, once under-coordinated Cu atoms of the Cu step-edge sites are anchored by dissociated O* from $CO_2$, the specific sites will be involved in irreversible surface reconstructions. The terminated oxygen of Cu step-edge sites may also react with neighboring CO adsorbates or gaseous $CO_2$ molecules under ambient $CO_2$(g) conditions, and the produced carbonate species (–$CO_3$) could participate in the surface morphology alterations. We found more roughened step-edge geometries after $CO_2$(g) evacuation, compared to the vicinal Cu surface after CO(g) evacuation, as shown in Supplementary Fig. 4.

## 2. Supplementary Figures

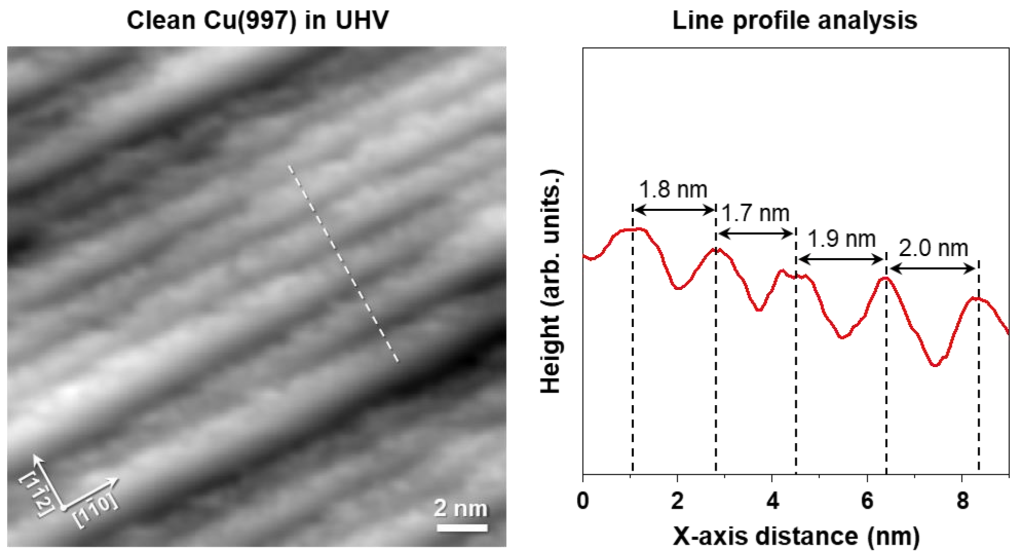


**Supplementary Fig. 1 |** Enlarged STM image of a clean Cu(997) surface in UHV (left) [$V_s$ = 1.00 V, $I_t$ = 340 pA]. A representative line profile analysis for step corrugations across the stepped morphology (right).

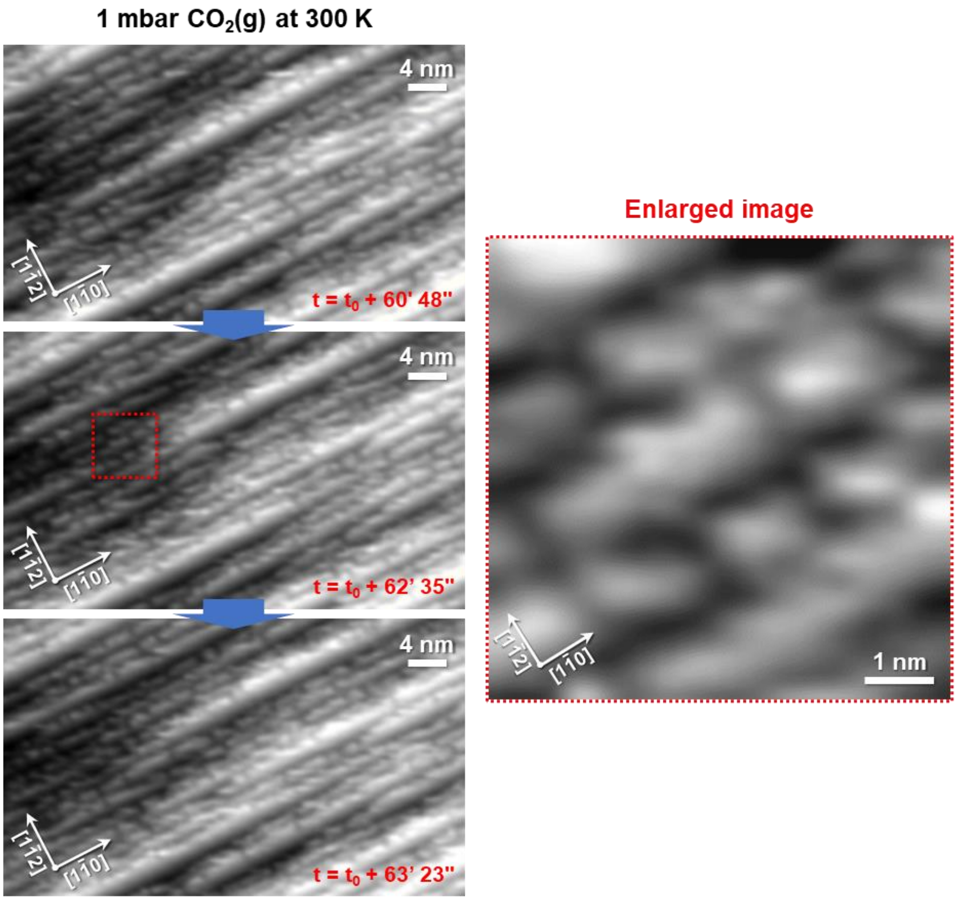


**Supplementary Fig. 2 |** AP-STM images on the Cu(997) surface under 1 mbar $CO_2$ gas (T = 300 K). Time-lapse observations show the evolution of Cu nanoclusters during the $CO_2$ dissociation on the stepped Cu surface [$V_s$ = 1.32 V, $I_t$ = 170 pA]. An enlarged image emphasizes a clear picture of the step-broken clustering phenomenon.

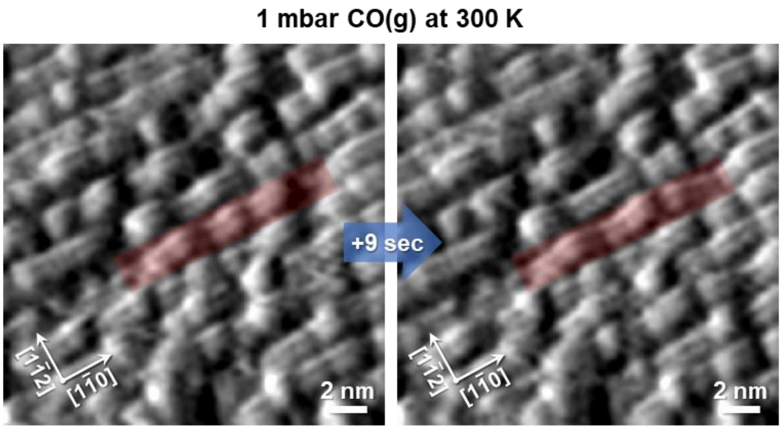


**Supplementary Fig. 3 |** AP-STM images on the Cu(997) surface under 1 mbar CO gas (T = 300 K). The discernible Cu nanoclusters evolution has lateral displacements along the direction of [1 –1 0] within 9 seconds. A red rectangle on the AP-STM image highlights the modulated local surface morphology [$V_s$ = 1.10 V, $I_t$ = 190 pA].

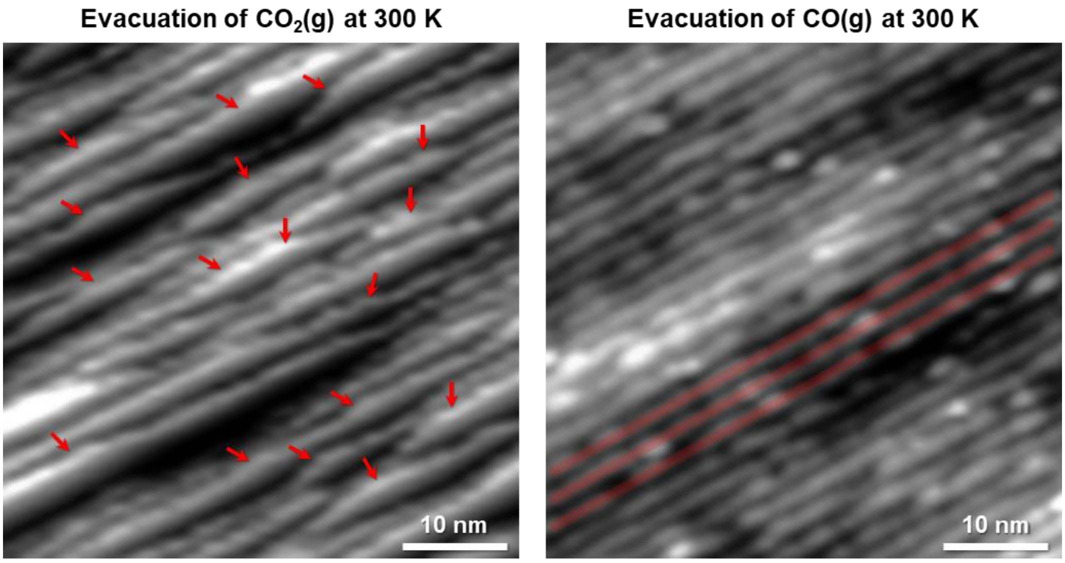


**Supplementary Fig. 4 |** AP-STM images on the Cu(997) surface after reaction gas evacuation conditions (T = 300 K). We found the remaining disordered structures by the influence of atomic O interactions after $CO_2$ dissociation (left). Red arrows indicate the reconstructed areas after the evacuation of $CO_2$(g) [$V_s$ = 1.28 V, $I_t$ = 160 pA]. The CO-induced clustering of Cu atoms is not observed after the CO(g) evacuation (right). Each step-width measured between Cu steps on the Cu(997) surface resembles the original vicinal morphology in UHV. Uniformly arranged red lines in the topographic image indicate the step boundaries [$V_s$ = 0.94 V, $I_t$ = 130 pA].

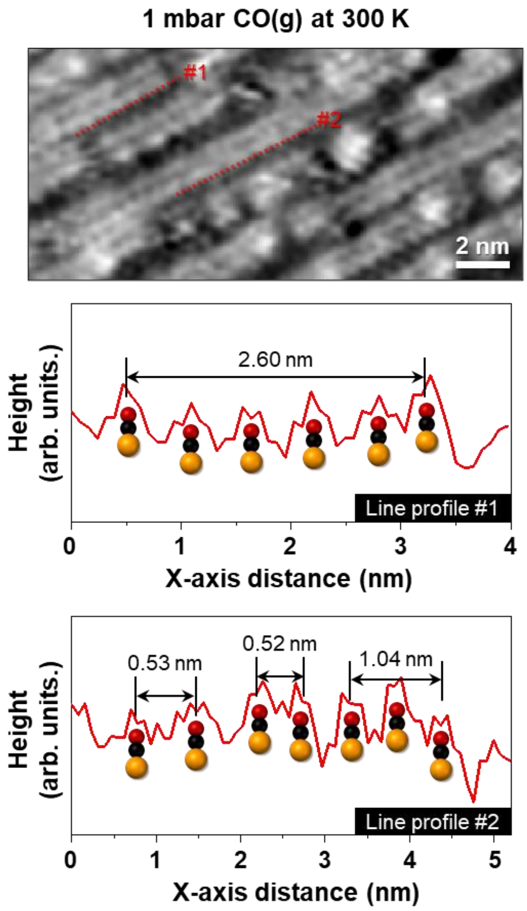


**Supplementary Fig. 5 |** A captured topographic image during time-lapse AP-STM observations under 1 mbar CO gas (top). We analyzed the atomic corrugation details from the differentiated AP-STM image to confirm CO-Cu sites at the step-edges. The indicated analysis results (bottom) display the measured peak-to-peak distances between illustrated CO-Cu corrugations.

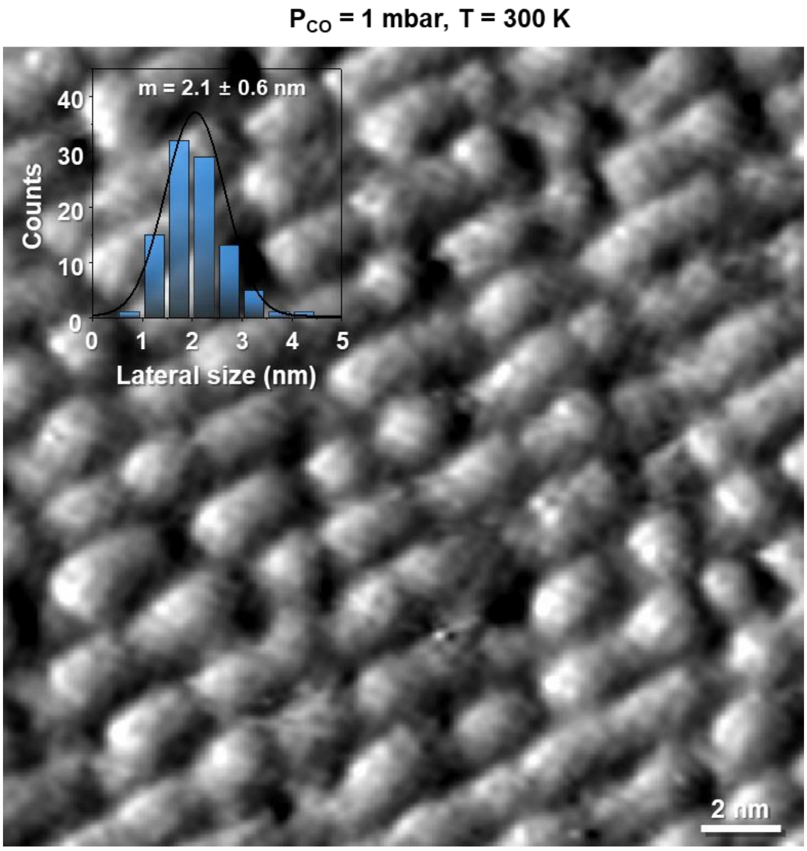


**Supplementary Fig. 6 |** Atom-resolved AP-STM image on the Cu(997) surface under 1 mbar CO gas. The histogram analysis (inset) from the topographic image shows that the characterized Cu nanoclusters have an average lateral size of 2.1 ± 0.6 nm.

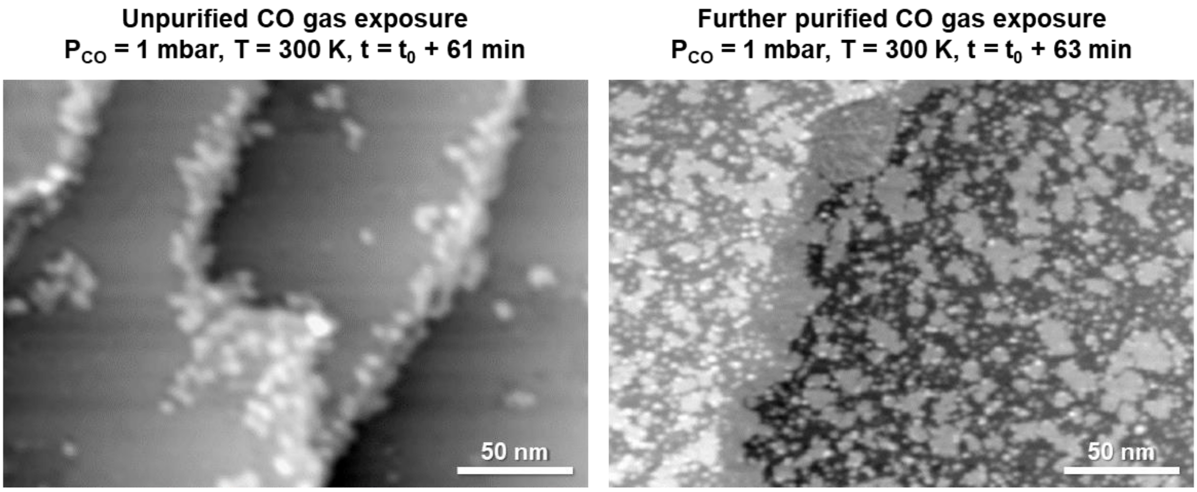


**Supplementary Fig. 7 |** The recorded AP-STM images on the Cu(111) surface after gas exposures to unpurified CO(g) or further purified CO(g) environments, respectively. The accumulated $Ni(CO)_4$ compounds are mainly well-characterized at step-edge sites (left). In contrast, the evolved $Cu_x(CO)_y$ clusters are widely formed across the terraces on the Cu(111) surface by CO-driven surface reconstruction (right).

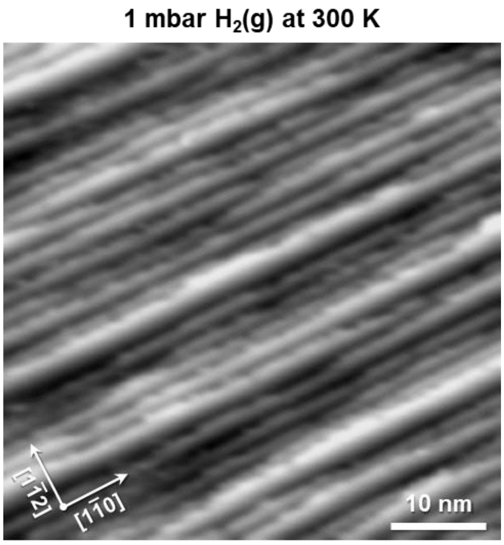


**Supplementary Fig. 8 |** AP-STM image on the Cu(997) surface under 1 mbar $H_2$ gas. The observed vicinal Cu surface had no surface reconstruction at 300 K. We confirmed that the residual $H_2$(g) in the UHV chamber did not affect our AP-STM experiments under gaseous environments.

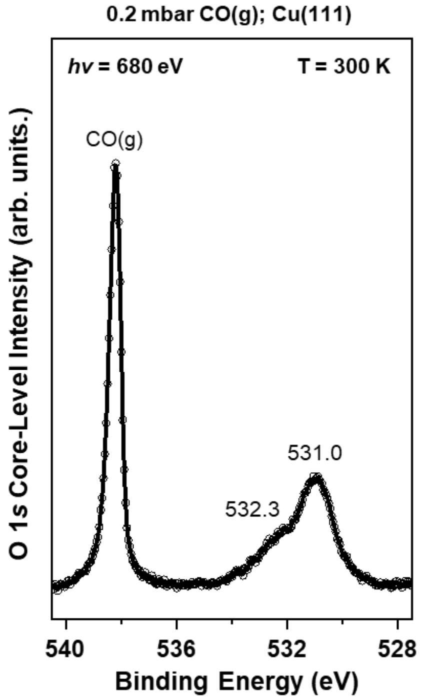


**Supplementary Fig. 9 |** The collected O 1*s* core-level spectrum of Cu(111) surface under 0.2 mbar CO(g) condition (*hv* = 680 eV; T = 300 K).

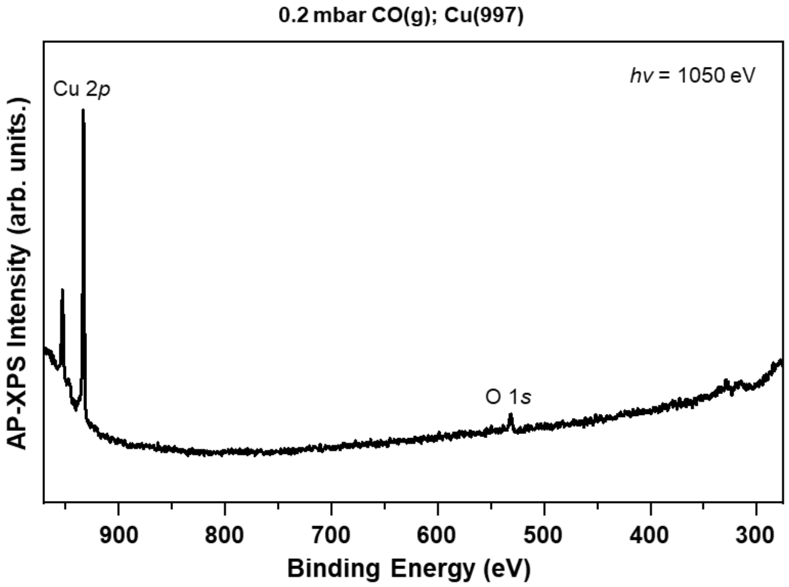


**Supplementary Fig. 10 |** The collected survey spectrum on the Cu(997) surface under the 0.2 mbar CO(g) ($hv$ = 1050 eV; T = 300 K).

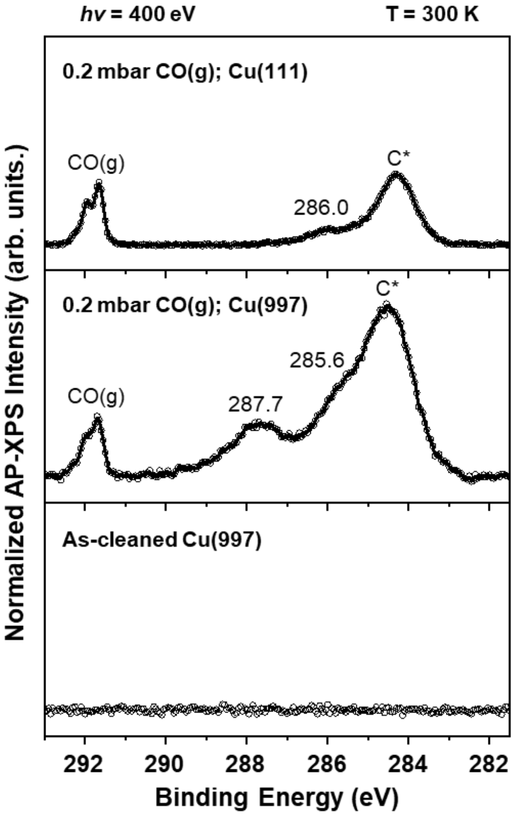


**Supplementary Fig. 11 |** Collected C 1*s* core-level AP-XP spectra from the Cu(997) and Cu(111) surfaces (*hv* = 400 eV; T = 300 K). As-cleaned Cu(997) surface in UHV (bottom) shows no spectroscopic detection of chemical species. We find that multiple peaks appeared in the C 1*s* core-level spectra of Cu(997) and Cu(111) after introducing 0.2 mbar CO(g) into the analysis chamber. The observed $sp^2$ (C=C) or $sp^3$ (C-C) carbon species at 284.2–284.3 eV would originate from the dissociation of CO or intrinsic carbon in bulk Cu lattice. The CO-induced morphology alterations on the vicinal Cu surface probably led to such upward segregation of buried carbon atoms at 300 K.

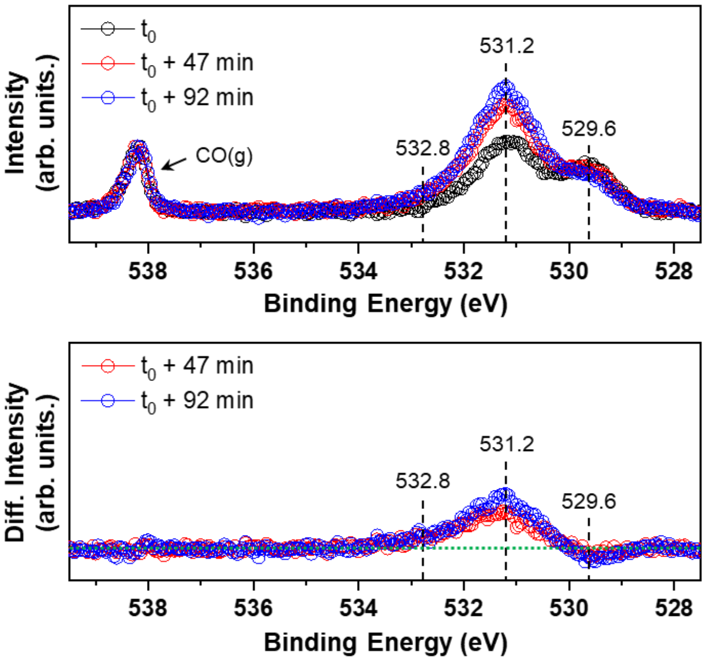


**Supplementary Fig. 12 |** Representative O 1*s* core-level spectra (top) and spectral subtraction plots (bottom) in time-lapse AP-XPS measurements ($h\nu$ = 680 eV) under the 0.2 mbar CO. The collected spectra at $t_0$ + 47 and $t_0$ + 92 min were processed by numerical subtraction of a spectrum at $t_0$ (initial reaction time). Measured peak intensities at 531.2 and 532.8 eV are gradually increased during the AP-XPS measurements, as plotted in signal intensity differences at $t_0$ + 47 and $t_0$ + 92 min from $t_0$ (bottom). The observed changes in peak intensities and spectral broadening features highlight convincing evidence for surface reconstruction under 0.2 mbar CO gas.

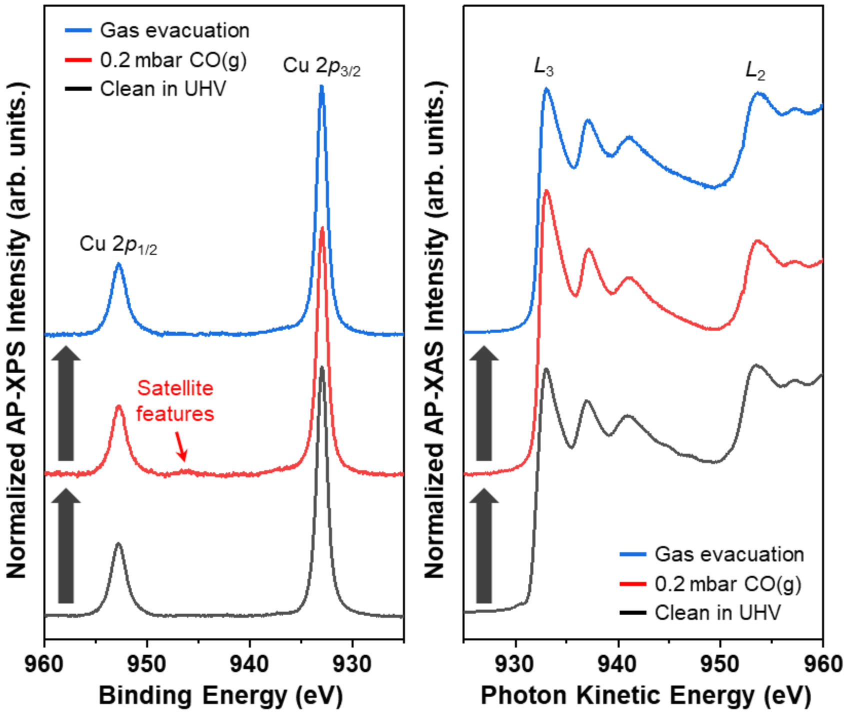


**Supplementary Fig. 13 |** Synchrotron-based X-ray photoelectron spectroscopy measurements for Cu $2p$ core-level states (left) and Cu $L_{2,3}$ absorption edge (right) on the Cu(997) surface. The escaped photoelectron and absorbed photon signal intensities were collected in analysis conditions of UHV, 0.2 mbar CO(g), and after gas evacuation ($hv$ = 1050 eV; T = 300 K). The plotted XPS results indicate no significant change in the oxidation state of Cu $2p_{3/2}$ and Cu $2p_{1/2}$. The spin-orbit couplings difference between Cu $2p_{3/2}$ and Cu $2p_{1/2}$ at 933.0 and 952.8 eV ($\Delta$ = 19.8 eV) was also kept under 0.2 mbar CO(g) conditions. We observed an unusual species at ~946.0 eV during AP-XPS measurements under 0.2 mbar CO(g), but the satellite features disappeared immediately in the Cu $2p$ core-level spectrum after gas evacuation. In plotted XAS results, the characterized Cu absorption edges show almost identical spectral shapes under different measurement conditions. The relative intensity changes in the $L_3$ edge of X-ray absorption spectra under 0.2 mbar CO(g) and gas evacuation conditions correspond to ~1.16 ($I_{red}/I_{black}$) and ~1.00 ($I_{blue}/I_{black}$), respectively.

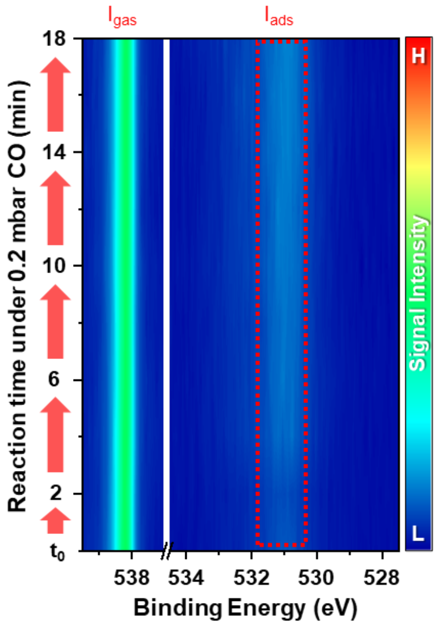


**Supplementary Fig. 14 |** A contour map analysis from time-lapse AP-XPS measurements on a Cu(111) surface under 0.2 mbar CO gas ($h\nu$ = 680 eV). The recorded signal intensity of CO(ads) was saturated at $t_0$ + 16 min after filling the analysis chamber with CO gas.

0.1 mbar CO(g) at 300 K

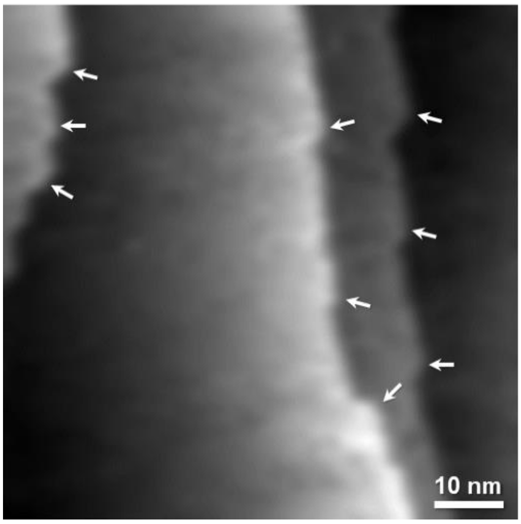


**Supplementary Fig. 15 |** AP-STM image on the Cu(111) surface under 0.1 mbar CO(g) at 300 K. Although we observed several CO-induced faceting results at step-edge sites, the terraces on the same surface kept their original morphologies without any restructuring process.

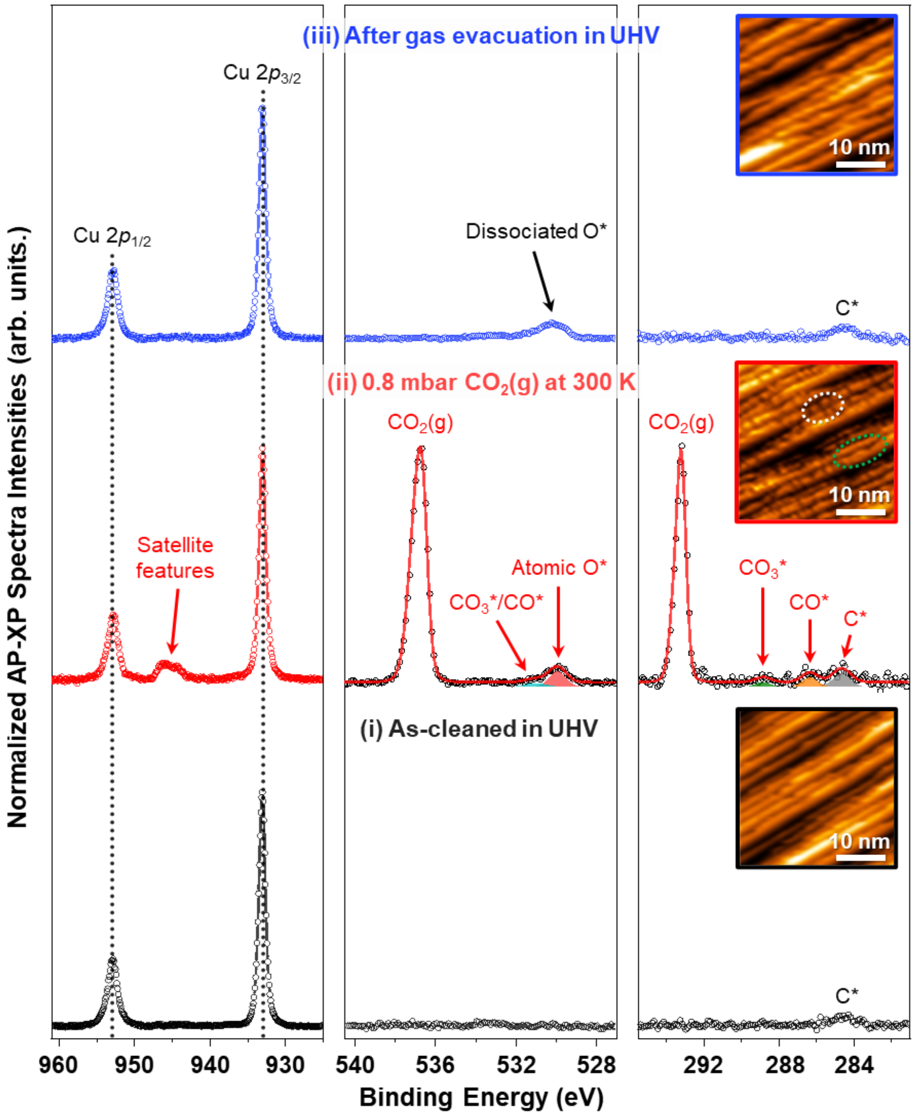


**Supplementary Fig. 16 |** Comparison observation results of $CO_2$ dissociation over vicinal Cu surfaces probed with AP-XPS and AP-STM. Collected Cu 2*p*, O 1*s*, and C 1*s* core-level AP-XP spectra in UHV (bottom; black line), 0.8 mbar $CO_2$(g) (middle; red line), and gas evacuation (top; blue line) conditions at 300 K ($h\nu$ = 1486.7 eV). The Cu(997) surface was exposed to 0.8 mbar $CO_2$(g) in the analysis chamber for 214 minutes before recording the plotted AP-XPS spectra (middle; red line). (**inset**) Corresponding morphology structures of the Cu(997) surface in UHV, 1 mbar $CO_2$(g) [$t = t_0 + 77$ min), and gas evacuation conditions. The displayed *in situ* AP-STM images were sequentially obtained in the same manner as **Figs. 1c-f** at 300 K.

## 3. Supplementary References


1 Rusponi, S., Boragno, C. & Valbusa, U. Ripple Structure on Ag(110) Surface Induced by Ion Sputtering. *Phys. Rev. Lett.* **78**, 2795-2798 (1997).

2 Giesen, M., Linke, U. & Ibach, H. Restructuring of the vicinal Cu(997) surface. *Surf. Sci.* **389**, 264-271 (1997).

3 Feenstra, R. M., Slavin, A. J., Held, G. A. & Lutz, M. A. Surface diffusion and phase transition on the Ge(111) surface studied by scanning tunneling microscopy. *Phys. Rev. Lett.* **66**, 3257-3260 (1991).

4 Bales, G. S. & Zangwill, A. Morphological instability of a terrace edge during step-flow growth. *Phys. Rev. B* **41**, 5500-5508 (1990).

5 Giesen, M. Step and island dynamics at solid/vacuum and solid/liquid interfaces. *Prog. Surf. Sci.* **68**, 1-154 (2001).

6 Kuipers, L., Hoogeman, M. S. & Frenken, J. W. M. Step dynamics on Au(110) studied with a high-temperature, high-speed scanning tunneling microscope. *Phys. Rev. Lett.* **71**, 3517-3520 (1993).

7 Kuk, Y., Chua, F. M., Silverman, P. J. & Meyer, J. A. O chemisorption on Cu(110) by scanning tunneling microscopy. *Phys. Rev. B* **41**, 12393-12402 (1990).

8 Wen, J. M., Chang, S. L., Burnett, J. W., Evans, J. W. & Thiel, P. A. Diffusion of Large Two-Dimensional Ag Clusters on Ag(100). *Phys. Rev. Lett.* **73**, 2591-2594 (1994).

9 Yang, X. *et al.* Low Pressure $CO_2$ Hydrogenation to Methanol over Gold Nanoparticles Activated on a $CeO_x/TiO_2$ Interface. *J. Am. Chem. Soc.* **137**, 10104-10107 (2015).

10 Salmeron, M. & Schlögl, R. Ambient pressure photoelectron spectroscopy: A new tool for surface science and nanotechnology. *Surf. Sci. Rep.* **63**, 169-199 (2008).

11 Waluyo, I. *et al.* Potassium-Promoted Reduction of $Cu_2O$/Cu(111) by CO. *J. Phys. Chem. C* **123**, 8057-8066 (2019).

12 Yamamoto, S. *et al.* Hydroxyl-Induced Wetting of Metals by Water at Near-Ambient Conditions. *J. Phys. Chem. C* **111**, 7848-7850 (2007).

13 Eren, B. *et al.* Identifying the catalyst chemical state and adsorbed species during methanol conversion on copper using ambient pressure X-ray spectroscopies. *Phys. Chem. Chem. Phys.* **22**, 18806-18814 (2020).
